# Large Pericardial Cyst: The Role of the DaVinci Robotic System

**DOI:** 10.7759/cureus.76994

**Published:** 2025-01-06

**Authors:** Joseph Okafor, Nikolaos Panagiotopoulos

**Affiliations:** 1 Cardiothoracic Surgery, Cleveland Clinic London, London, GBR

**Keywords:** da vinci robot, multimodality cardiac imaging, pericardial cyst management, robotic-assisted surgery, symptomatic pericardial cyst

## Abstract

Pericardial cysts are a rare but important differential diagnosis when considering mediastinal lesions. Cyst removal is commonly indicated for diagnostic purposes. Removal should also be considered in the context of infection and cyst growth, which could lead to compression of the right ventricle. We describe the case of a patient who underwent robotic-assisted excision of a pericardial cyst as well as the surgical management of such lesions.

## Introduction

A pericardial cyst is a rare clinical entity that is usually identified incidentally. They account for 7% of all mediastinal tumors and are an important differential when considering the nature of any mediastinal mass [1]. Surgical excision is typically indicated for diagnostic purposes when the etiology of the mass cannot be identified with cardiac imaging. Cyst removal is also indicated for therapeutic purposes in treating symptoms caused by inflammation, infection, or compression of surrounding structures [2]. The DaVinci Robotic System (Intuitive Surgery Inc., California, USA) is a surgical advance that can be used in the minimally invasive management of mediastinal pathology. Compared to open thoracic surgery, the DaVinci robot has several patient advantages, including shorter hospital stays, improved recovery, and fewer perioperative complications [3]. We describe the case of a patient who underwent robotic-assisted excision of a pericardial cyst as well as the surgical management of such lesions.

## Case presentation

A 29-year-old gentleman presented with a one-year history of unexplained exertional dyspnea, recurrent chest pains, and palpitations. The chest pains were sharp in nature and not related to exertion. On clinical examination, he was of athletic build and appeared comfortable at rest. On auscultation, he had normal heart sounds, no murmurs, and clear lung fields. His pulse rate was regular at 87 bpm, and his blood pressure was 121/87 mmHg.

Laboratory workup revealed a normal full blood count, liver function tests, and renal profile (Table 1). His troponin was not elevated. Inspection of the 12-lead ECG revealed normal sinus rhythm with no ST-segment or T-wave abnormalities. The QRS complexes were narrow, and there were no features of atrioventricular block.

**Table 1:** Laboratory workup

Laboratory workup		
Laboratory test	Result	Reference range
Hemoglobin	153	130-175 g/L
White cells	7.37	3.5-11 x 10^9/L
Platelets	275	150-400x10^9/L
Sodium	139	135-145 mmol/L
Potassium	4.9	3.5-5.1 mmol/L
Urea	7.7	1.7-8.3 mmol/L
Creatinine	96	59-104 umol/L
Glomerular filtration rate	>90	>90 mL/min
Bilirubin	11	0-20 umol/L
Alkaline phosphatase	47	40-129 IU/L
Alanine transferase	34	10-50 IU/L
Gamma-glutamyl transferase	24	10-71 IU/L
Albumin	46	34-50 g/L
High-sensitivity troponin T	5	<14 ng/L

The unremarkable chest X-ray showed clear lung fields and a normal cardiac silhouette. Transthoracic echocardiography revealed normal biventricular size and systolic function. In particular, right ventricular function was normal (TAPSE 1.9 cm, RV S' 13 cm/s). The inferior vena cava was non-dilated with >50% inspiratory collapse. Trivial tricuspid regurgitation was seen. The estimated pulmonary artery systolic pressure was 20 mmHg, and there were no echocardiographic features of pulmonary hypertension. An obvious extracardiac mass was not well visualized. There was no obstruction to the right heart filling.

**Figure 1:**
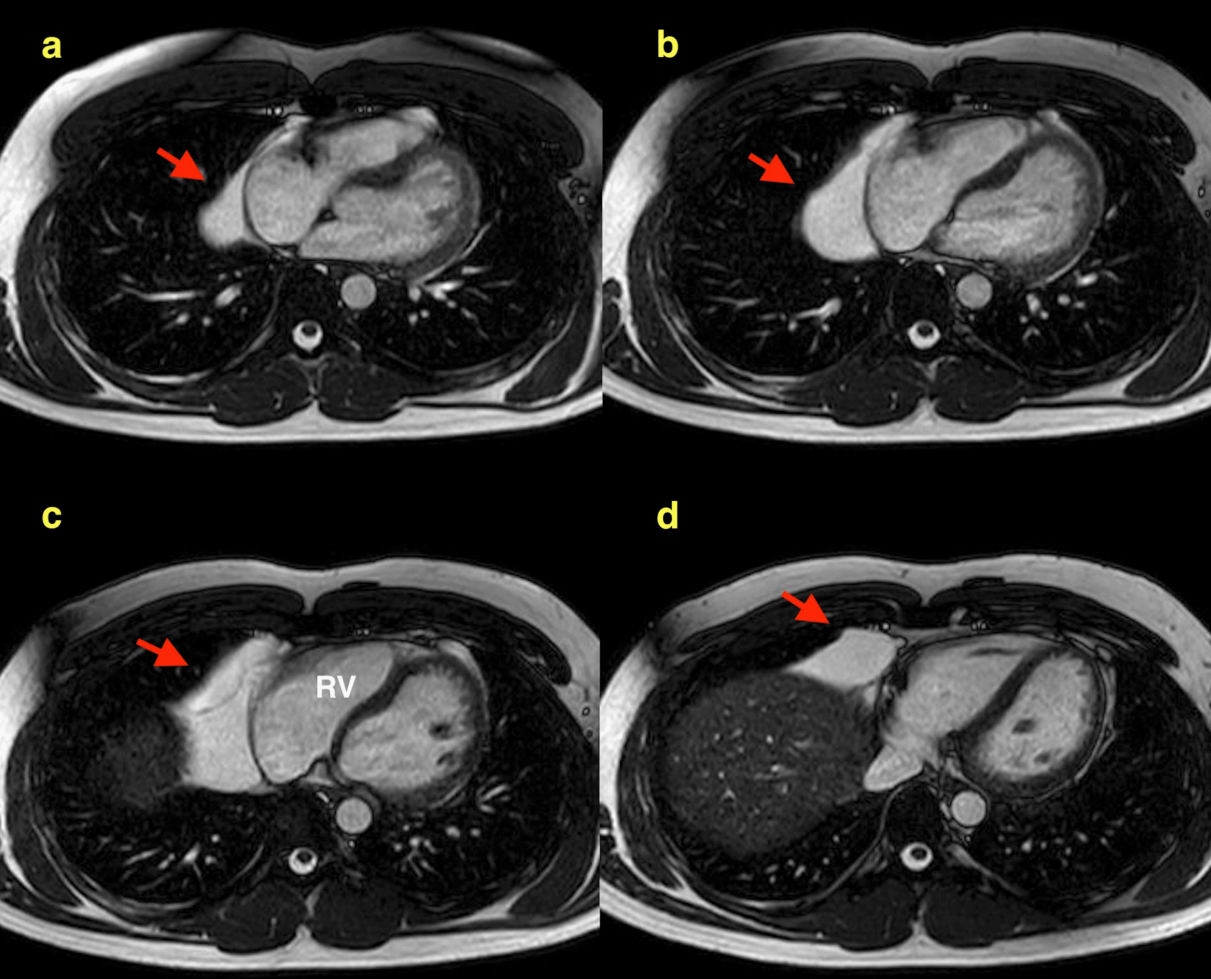
CMR imaging of the pericardial cyst (red arrows) displayed in the axial plane Sequential images (from a-d) highlight the cranial (a) to caudal (d) extent of the cyst. The cyst encases much of the right heart, including (c) the free wall of the RV. No tricuspid regurgitation or caval compression is seen. RV: right ventricle, CMR: cardiac magnetic resonance

He underwent cardiac magnetic resonance (CMR) imaging at 1.5 Tesla. This revealed normal biventricular volumes with good systolic function. The left ventricular ejection fraction was 64%, with no significant valvular pathology. A large 9x3 cm pericardial cyst was identified, which appeared to be occupying the lateral aspect of the right ventricle and causing minor external compression (Figure 1).

**Figure 2:**
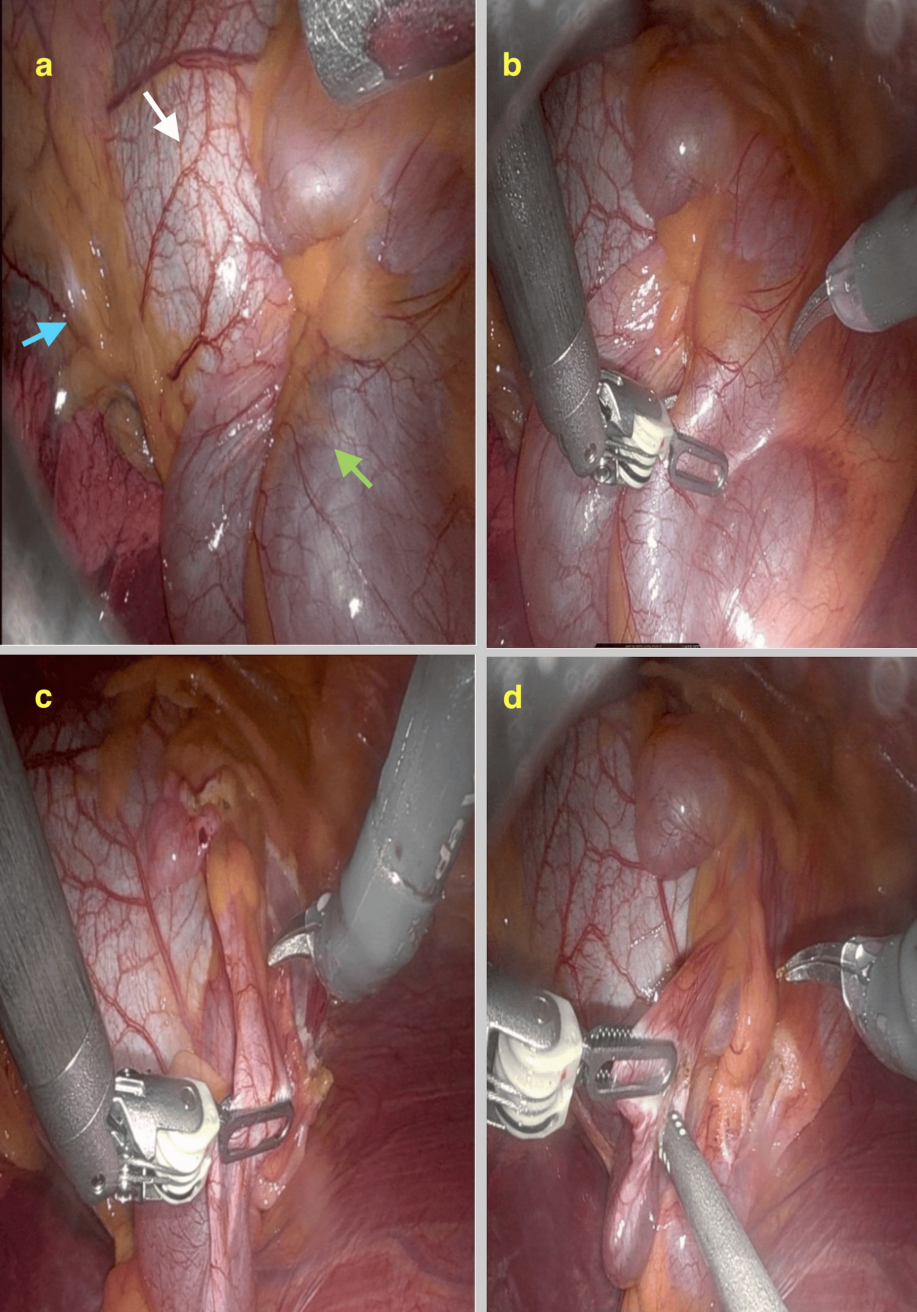
Panel of intraoperative images Excellent visualization is afforded with the DaVinci Robotic System (a). Prior to removal, the large multiloculated cyst is seen on the right-hand side (green arrow), closely adherent to the pericardium (white arrow). The phrenic nerve is carefully identified (blue arrow). (b) Bipolar graspers are used to detach the cyst from the rest of the pericardium, and (c) careful cutting away of the bulk of the cyst is performed. This allows insertion (d) of the suction port to aspirate the clear cystic content.

The DaVinci Robot Xi was used to resect the pericardial cyst. The patient was placed in a supine position with the right side tilted upwards to 45 degrees. Under general anesthesia and with a double-lumen endotracheal tube to isolate the right lung, 3 x 8 mm incisions were performed to facilitate the introduction of the camera, monopolar scissors, and a bipolar grasper. An additional 12 mm incision allowed the introduction of the assistant port. The chest cavity was insufflated with carbon dioxide. As per the CMR, the multiloculated cyst was identified above the pericardium and lateral to the right ventricle (Figure 2). The phrenic nerve was identified and protected from injury. Drainage was followed by a complete resection of the whole cyst while maintaining the integrity of the pericardium. Cloudy, yellow, gelatinous fluid was aspirated from the cyst. Minimal blood loss from the procedure was observed, and the total operative time was less than one hour. A right-sided chest drain was inserted and then removed the same day. Both lungs were well expanded on the post-drain removal chest X-ray. The postoperative course was uneventful, and there were no clinical signs of phrenic nerve palsy. The patient was successfully discharged within 24 hours.

Microscopic fluid analysis revealed cellular contents and cytospins with lymphocytes, macrophages, and mesothelial cells. The appearances favored reactive inflammatory changes. Histopathological analysis showed a cyst lined by cytologically bland cuboid epithelial cells with the wall showing fibrosis and patchy, non-specific chronic inflammation. Overall, the features favored benign mesothelial cysts. At a three-month follow-up, the patient remained well and free of his previous chest pain and palpitation symptoms. Computed tomography (CT) of the thorax revealed no reaccumulation of the pericardial cyst and no pleural or pericardial effusion. At the 12-month follow-up, the patient was physically active and reported no further chest pains.

## Discussion

Pericardial cysts are a rare benign mediastinal mass ordinarily detected in the asymptomatic individual. They are an important differential diagnosis when considering the etiology of mediastinal lesions and account for 7% of all mediastinal tumors [1]. An incidence of approximately one in 100,000 patients has been reported [4]. Males and females are equally affected and frequently detected in the third or fourth decade. Most lesions are congenital due to incomplete fusion in embryogenesis, leading to herniation in the pericardial sac and diverticulum formation. Cysts may also be acquired following cardiac surgery, pericarditis, trauma, tuberculosis, or hemodialysis [5,6]. In 50-75% of cases, patients with pericardial cysts are asymptomatic [2]. When symptoms do occur, clinical presentation is related to the cyst's complications. Compression of the right heart may cause right-sided heart failure or superior vena cava obstruction [7-10]. Cardiac tamponade secondary to rupture into the pericardium has been reported [11]. Other presentations include recurrent cough, pneumonia, retrosternal pressure, chest pain due to pericarditis, syncope, and palpitations secondary to atrial fibrillation [12-16]. The size of cysts varies, with an average diameter of 5.4 cm being reported [17]. However, the excision of up to 28 cm lesions has also been described [18]. Typically, they contain clear fluid. Cysts may be visible on transthoracic echocardiography. However, cross-sectional imaging such as CMR is valuable for delineating the full extent of the cyst, its relationship, and its attachment to neighboring structures and aiding in providing a differential diagnosis. A definitive diagnosis is made following histopathological examination following cyst removal. As in this case, removal is commonly indicated for diagnostic purposes. Removal should also be considered in the context of infection and cyst growth, which could lead to compression of the right ventricle. Prior to the advent of video-assisted thoracoscopic surgery (VATS), surgical excision was performed via open thoracotomy. Such a minimally invasive approach is now favored over open thoracotomy due to earlier mobilization and less pain in the postoperative period [3]. Robotic-assisted resection offers several advantages over the VATS approach. The surgeon has greater freedom of movement, which is digitally and mechanically optimized. This results in reduced hand tremors and increased precision, particularly in operations that require great delicacy, such as this one. Disadvantages include the increased upfront cost required to obtain the robotic system. Additionally, the surgeon lacks tactile feedback, which may affect the accurate palpation of tissues.

## Conclusions

Pericardial cysts are a rare but important differential diagnosis when considering mediastinal lesions. The patient is often asymptomatic, but clinical presentation may vary depending on the effect of compression on surrounding structures or inflammation. Multimodality imaging with echocardiography and cross-sectional methods is essential in assessing size and character and excluding complications from the cyst. Removal is often necessary for diagnosis and symptomatic purposes. When indicated, surgical excision with a robotic system has several benefits and should be considered the preferred approach. Further research and evaluation of larger case series are required to fully understand the incremental benefit of robotic-assisted pericardial cyst removal compared to other surgical approaches.
